# Supplementary figures and images for: The ciliary kinesin KIF7 controls the development of the cerebral cortex by acting differentially on SHH signaling in dorsal and ventral forebrain
Source: eLife. 2025 Sep 16;13:RP100328. doi: 10.7554/eLife.100328 (PMC12440355; doi:10.7554/eLife.100328)

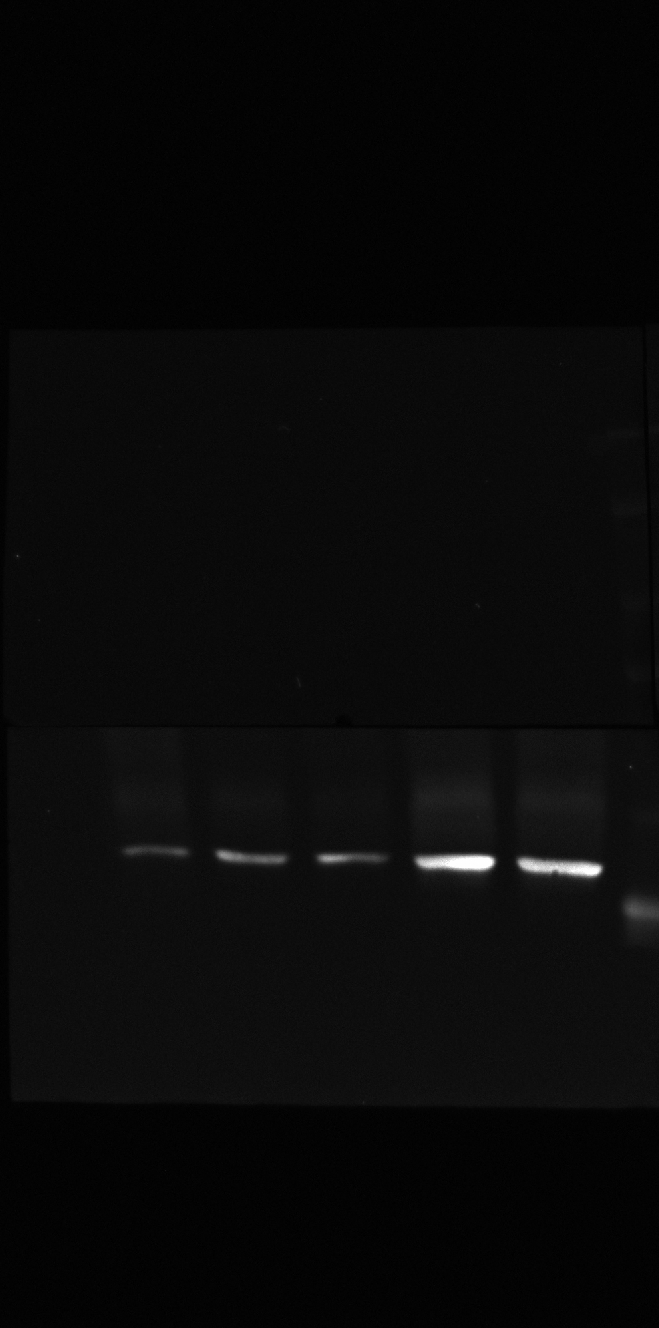

Supplement: Figure 2—source data 1. [file elife-100328-fig2-data1.zip › W blot actin bis original uncropped image 2.tif]

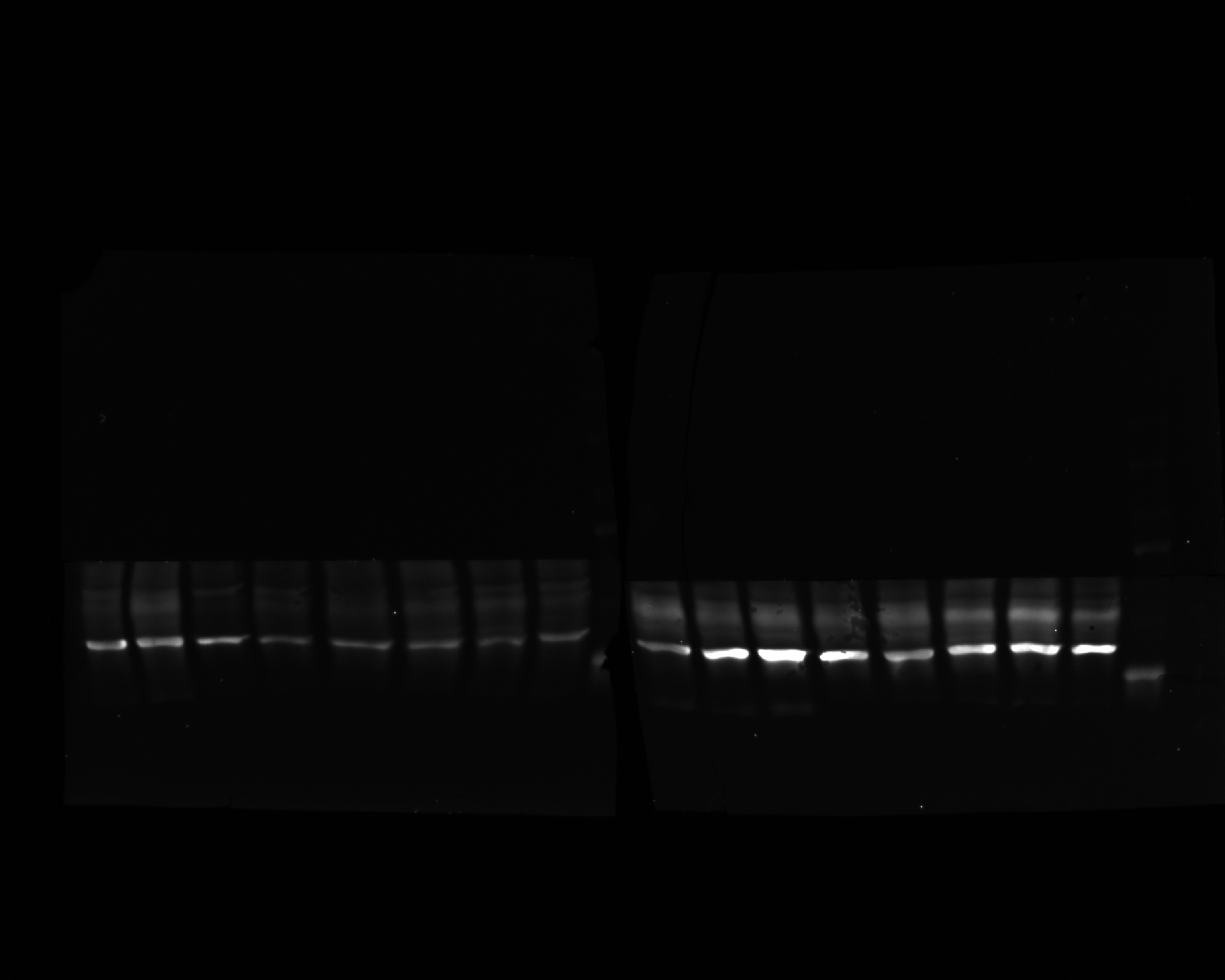

Supplement: Figure 2—source data 1. [file elife-100328-fig2-data1.zip › W blot actin originaluncropped image.tif]

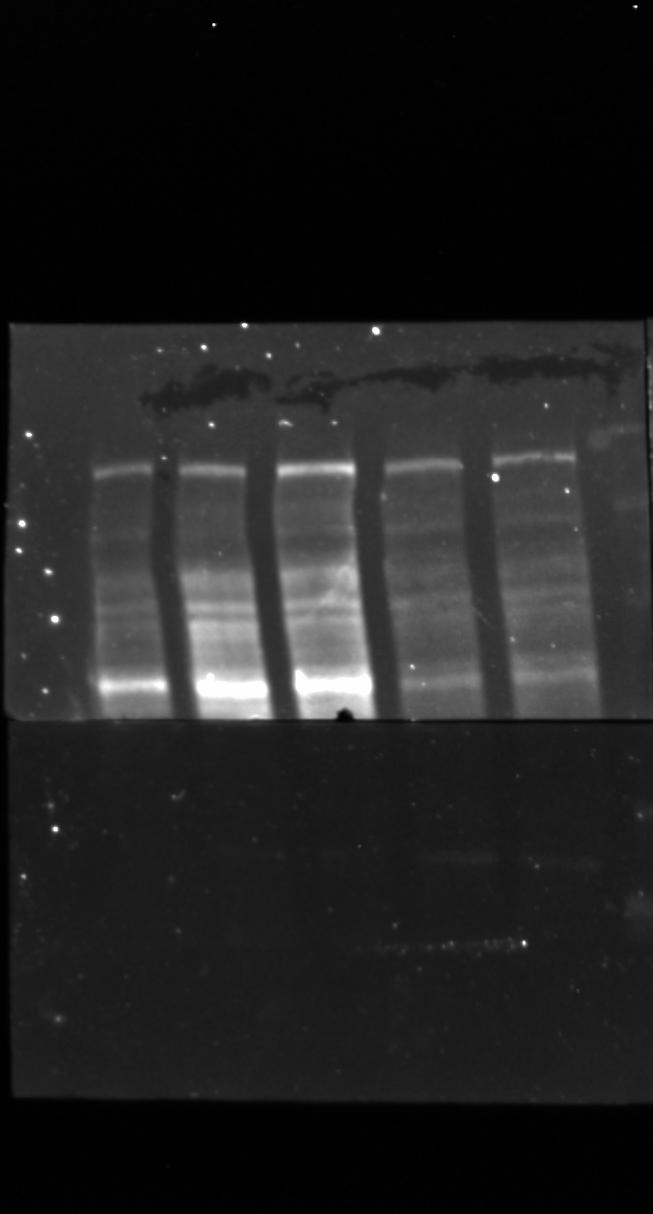

Supplement: Figure 2—source data 1. [file elife-100328-fig2-data1.zip › W blot GLI3 bis original uncropped image 2.tif]

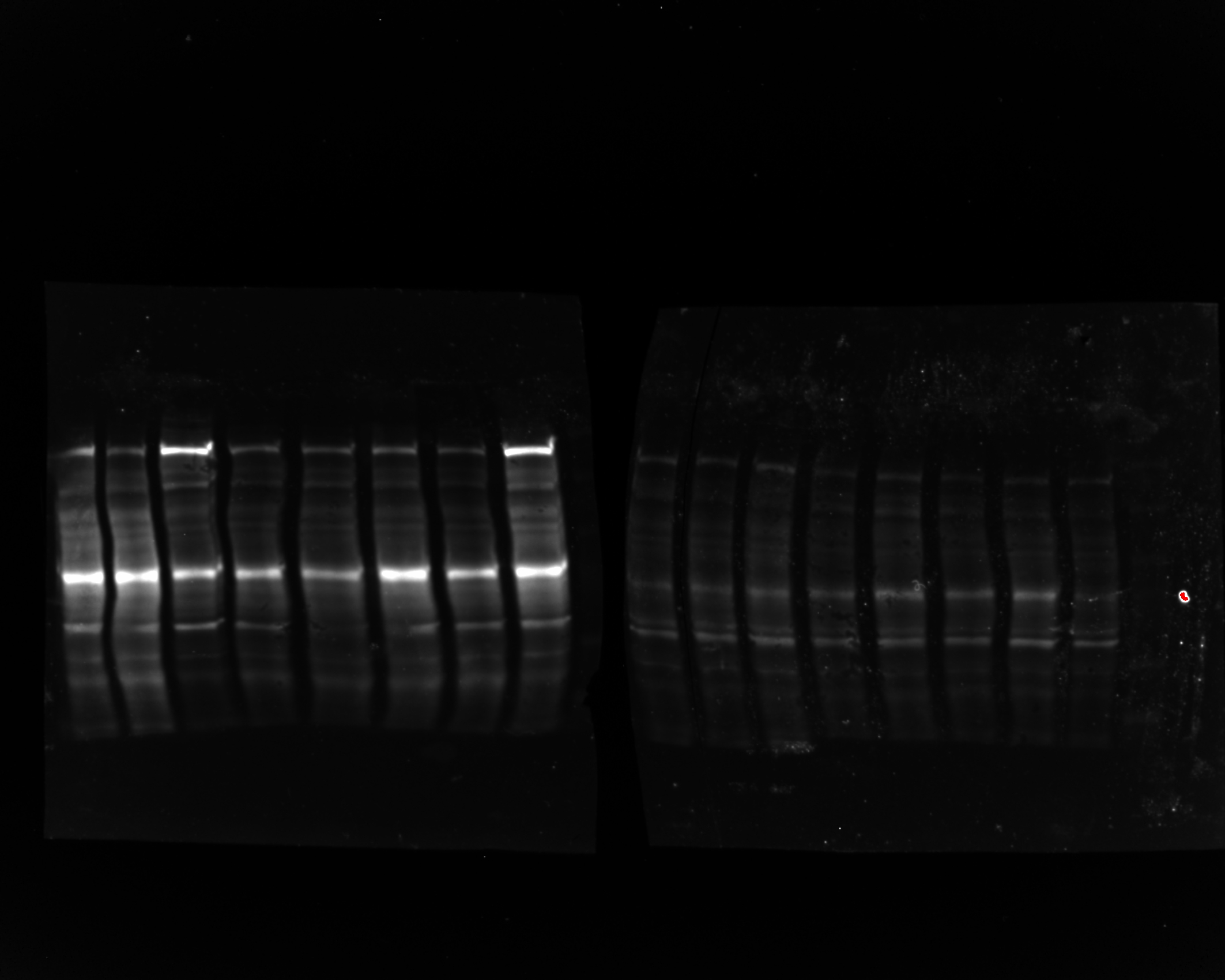

Supplement: Figure 2—source data 1. [file elife-100328-fig2-data1.zip › W blot GLI3 original uncropped image.tif]
