# Supplementary material for: The ciliary kinesin KIF7 controls the development of the cerebral cortex by acting differentially on SHH signaling in dorsal and ventral forebrain
Source: eLife. 2025 Sep 16;13:RP100328. doi: 10.7554/eLife.100328 (PMC12440355; doi:10.7554/eLife.100328)
Supplement: Figure 2—source data 2. — Note that samples from heterozygous animals were not presented in Figure 2. [file elife-100328-fig2-data2.pdf]

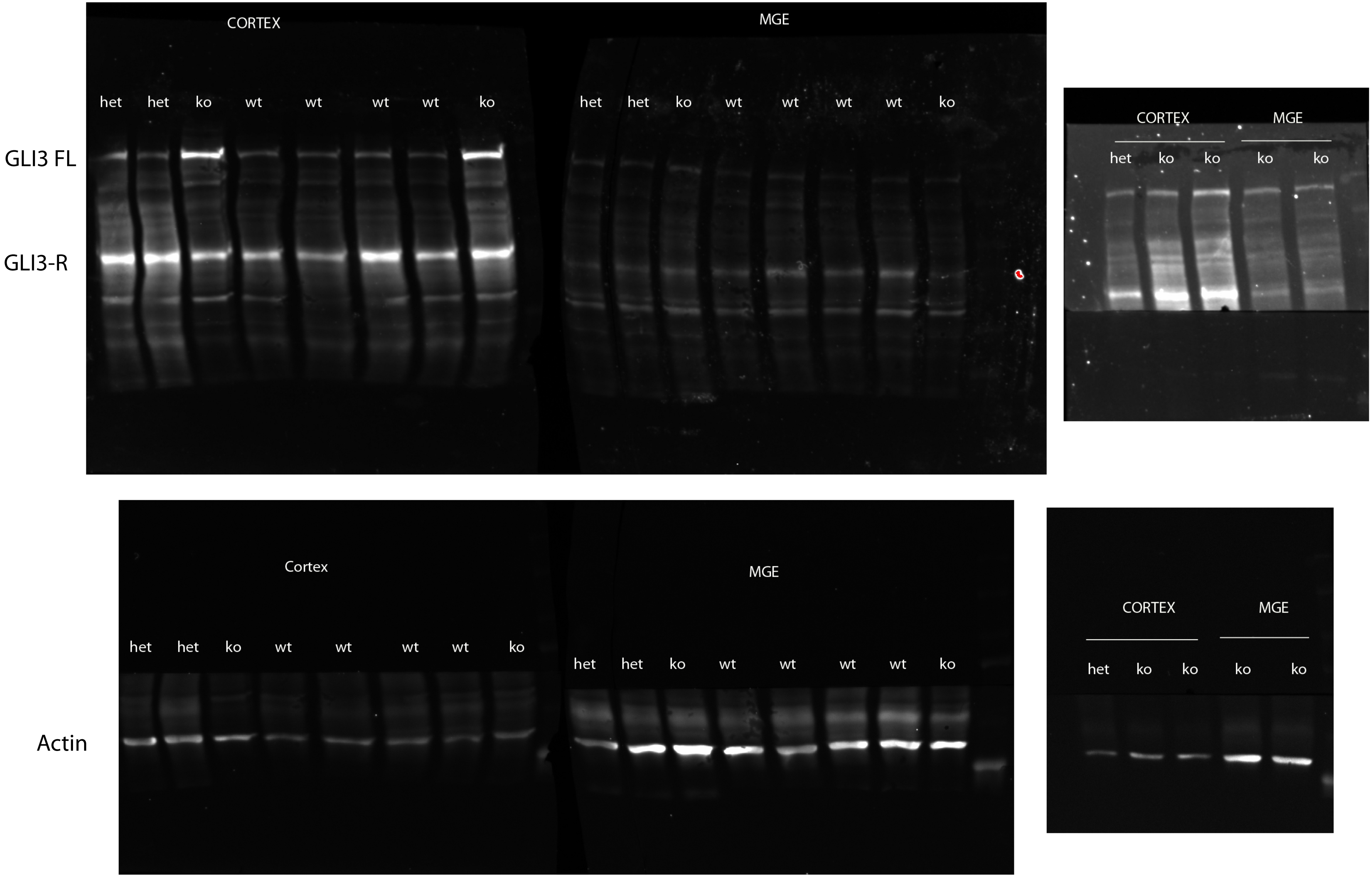


Figure 2-source data 2. Original membranes corresponding to Figure 2, indicating the genotype of samples and relevant bands. Note that samples from heterozygous animals were not presented in Figure 2A and not quantified.
